# Proceedings of the Michigan State Dental Association

**Published:** 1870-03

**Authors:** Geo. H. Mosher


### Proceedings of Societies.

### PROCEEDINGS OF THE MICHIGAN STATE DENTAL ASSOCIATION.

The fifteenth annual meeting of the Michigan State Dental Association convened pursuant to adjournment, at the Russell House, Detroit, at 12 o'clock M., Tuesday, October 12th, 1869.

The President and Vice-President being absent, Dr. Holmes of Grand Rapids was called to the Chair. A small portion of the routine business was gone through with, after which the Association adjourned until 2 P. M., at which time the Association met, and proceeded to take up the regular order of business; first in order being the reception of proposals for membership, a number of which were referred to the examining committee.

The minutes of the last meeting were then read and after correction approved.

| | |
|---|---|
| The Treasurer reported receipts | $139 90 |
| Disbursements | 78 52 |
| Leaving a balance on hand of | $ 61 38 |

Report referred to the examining committee.

The Secretary submitted his annual report, showing that at the last meeting there were five initiations, and that the amount of money received by him is as follows:

| | |
|---|---|
| From retiring Treasurer | $20 90 |
| For dues | 34 00 |
| On behalf of memorial committee | 85 00 |
| Total | $139 90 |
| Warrants issued | 12 75 |
| Balance paid to new Treasurer | $127 15 |

The Secretary expressed regret that a more general interest was not taken in the affairs of the Association, and hoped that in the future there would be a better attendance upon its meetings, and a more hearty interest in its affairs manifested.

The report was referred to the examining committee. The Secretary's bill for incidental expenses—$13 45—was also referred to the same committee.

An order for $13 45 was drawn in favor of Dr. Jackson, ex-Secretary, for expenses incurred during his term.

In the absence of Dr. Crooks, Dr. Douglass was appointed to fill his place on the Examining Committee, when that committee retired for the examination of candidates, reporting favorably upon the names of G. E. Corbin, of St. Johns, Geo. I. Holmes, of Battle Creek, J. W. Storms, of Jonesville, and W. P. Cutler, of Ionia. Report accepted, and the gentlemen were elected members by ballot. The Committee also reported favorably on the Secretary's bill, and recommended its payment.

Adjourned until 9 o'clock A. M., of the following day.

In the evening, an informal social meeting of the association was held, for the interchange of sentiment and information.

Wednesday, 9 o'clock A. M., October 15th, the Association met, pursuant to adjournment, Dr. Watling, of Ypsilanti, in the Chair. Dr. C. B. Porter, of Ann Arbor, was elected an active member of the Association by acclamation.

On motion of Dr. Holmes, of Grand Rapids, a committee was appointed to revise and codify the constitution, by-laws and rules of order. The following gentlemen were appointed as such committee: E. S. Holmes, Grand Rapids; G. R. Thomas, Detroit; C. B. Porter, Ann Arbor, and J. H. Warner, St. Clair.

The Association next proceeded to elect officers for the ensuing year, with the following result:

President, E. S. Holmes, Grand Rapids.

Vice President, R. S. Bancroft, Romeo.

Secretary, G. H. Mosher, Jackson.

Treasurer, J. A. Watling, Ypsilanti.

Executive Committee, G. W. Stone, Albion ; J. A. Watling, Ypsilanti, and G. P. Holmes, Battle Creek.

After the election of officers was concluded, the President elect was conducted to the Chair.

He thanked the Association for the honor conferred upon him, but confessed to some embarrassment in being called to a position he had not sought. He would endeavor to do his duty, but looked to the members for their assistance, without which he could not hope to succeed as their presiding officer. In discussions, he expected members would confine themselves to the question at issue, and if he exercised his privilege of calling them to order, it would only be for the good of the Association. If preliminaries were avoided, more business could be accomplished in one day than otherwise in two or three. The President again thanked the Association for the honor, and the regular order of business was resumed.

After some discussion, Jackson was selected as the place for holding the next annual meeting.

Dr. Thomas, from the Examining Committee, announced the report of the Secretary correct, and the report of the Treasurer incorrect, the latter having overcharged himself $6 00. The report was corrected, and, on motion, $5 00 refunded to the Treasurer, he having contributed a special amount to defray the expenses of the Memorializing Committee.

The balance in the Treasurer's hands, $50 38, was ordered to be paid into the general fund.

The question of Sensitive Dentine was taken up for consideration.

Dr. Corbin, of St. John, said in treating patients he had reference to their temperament. If the cavities were numerous and the patient sensitive, he generally found cotton saturated with creosote quite effective. If chloroform was used, a coating of gum mastic, with cotton, would protect the dentine from the action of the saliva, as well as prevent evaporation, for weeks. In one instance he had found the cotton remaining in the cavity several months after he had applied it as mentioned, and without further decay to the tooth. Chloride of zinc was more prompt, but painful, and apt to destroy the life of the dentine.

Dr. Douglass, of Romeo, said that he had been accustomed, recently, to diet his patients who were subject to sensitive dentine, and found the result very satisfactory. In connection with this treatment, he uses carbonate of lime ; in dieting, he uses graham bread, taking little or no drink at meals, tea without milk or sugar, and no sugar, except in food; to drink nothing until meals have digested, and then to drink three or four times of water before the next meal.

Dr. Thomas, of Detroit, thought that the remedies first suggested, by Dr. Corbin, were ineffectual, and preferred chloride of zinc, though the latter must be used with care. Arsenic with creosote is sometimes used, but it destroys the pulp, and should therefore be abandoned. He thought, also, that a sharp excavator was the best tool for dealing with sensitive dentine, though remedies for deadening the pain may be employed with advantage. Chromic acid is frequently used, but is somewhat dangerous unless the greatest care is used.

Dr. Field, of Detroit, has used the various remedies, except arsenic, which, like the boy who trimmed the dog's tail close to his ears, was sure to kill the tooth. He greatly favored the free use of the excavator, and is very favorable to the use of creosote, which he considers the little giant of them all.

Dr. J. H. Warner, of St. Clair, said there was nothing like sharp instruments. He liked carbolic acid, for if the cavity could be reached he believed there was something in it that operated well. Arsenious paste, chloride of zinc,etc., were all right if removed at exactly the right time. A bold hand was needed to do the cutting, yet without constitutional treatment all remedies might be only half-way.

Dr. Corbin regarded sharp instruments as a foregone conclusion. He believed the pulp fiber analogous to nerve matter; creosote gradually rendered the nerves sensitive toward the pulp.

Adjourned to 2 o'clock P. M.

*Afternoon Session.*

Dr. Crooks, of Birmingham, deprecated the use of arsenious acid as most dangerous to the teeth, unless possibly in case of an aged patient, and then it should only be used when the case was an extreme one. The use of this acid he thought to be so fatal to the teeth that he would generally advise extraction of the tooth in preference to its use. The use of chloride of zinc he favors, and uses extensively in his practice, with favorable results.

The next subject for discussion was "Alveolar Abscess."

Dr. Thomas, in opening the discussion, remarked upon the various causes that induce abscess or ulceration,and contended that after the abscess had once formed it was impossible to save the tooth. He held that abscess or ulceration never occurred until the tooth was dead. He differed from the views held by some gentlemen of the profession, that a tooth was never really dead until extracted.

Dr. Crooks thought trephining might be made to answer, removing the portions affected. He would cut through the alveolar process and remove the diseased portion of the jaw or root of the tooth, but it would be a painful operation and it might be better to extract the tooth at once. If ulceration has lasted two or three months, the latter is the only wise course.

The order of business was suspended to take up the case of A. F. Douglass, of Fenton, an applicant for membership. A motion to suspend the rule requiring applicants to have been six years in practice was made. After some discussion it was resolved to adhere to the rules, and Dr. Douglass was not admitted; but he, as well as other members of the Dental or Medical profession, were invited to take seats in the Association.

The question of Alveolar Abscess was resumed.

Dr. Douglass, of Romeo, said that in cases where there was little or no pain, and no outward inflammation, his plan was to clean out the nerve canal, washing it with creosote, and then sponging creosote into the abscess till it emerged through the fistulous opening. He then proceeded, at the same sitting, to fill both root and crown with gold.

Dr. Holmes, of Grand Rapids, thought that a great number of cases that came under the notice of Dentists could be cured if carefully and assiduously treated. He greatly valued a natural tooth. He thought that when there was any hope of saving the natural teeth it was the duty of the Dentist to do all he could to accomplish that object. He hoped that the time would come when the making of artificial teeth would be no more a part of the Dentist's profession than the making of artificial limbs was of the surgeon's.

Dr. Bancroft, of Romeo, suggested that in difficult cases a good deal might be gained by pursuing vigorous measures for a short time, and then suspending operations long enough to allow nature to act in the aid of art.

Dr. Douglass, of Romeo, advocated the application of chloride of gold, provided great care was taken in its use.

The case of Dr. Kibbee, of Port Huron, an applicant for admission to the Association, was taken up, and a very animated discussion ensued touching the matter. It was finally adjourned one year

Dr Holmes, of Battle Creek, thinks alveolar abscess can be cured; were we to extract all teeth which are ulcerated, we should produce serious injury upon our patients; has not had a failure in eight years; treats through the nerve canal invariably; cleans the cavity well, and applies remedies to assist nature in effecting a cure. It will require a longer or shorter time, dependent upon the health of the patient.

Dr. Warner, of St. Clair, always opens through the canal, breaking up the sac. If the case is obstinate, treats through the alveolus, and generally finds the case yields to such treatment.

Dr. Thomas presented the case of a little girl, twelve years of age, with a necrosis of the jaw, from which he had taken two teeth and a piece of necrosed bone. The gentlemen present examined the case carefully, and spent the remainder of the afternoon session in general remarks of a conversational nature, during which a motion was made to suspend the rules making it necessary for practitioners to go through a definite course of study and graduating before they could be admitted into the Association, which called forth the almost unanimous voice of the members of the Association against "letting down the bars" in any case, thus practically lowering the standard of qualification. The conservative tone of the remarks made argues well for the high position Dentistry is to take among the professions of our State.

Adjourned till half-past 7 o'clock in the evening.

*Evening Session.*

The next subject for discussion, "Inflammation of the Dental Pulp and Dental Periosteum, was taken up."

Nine o'clock Thursday morning was set as the hour for clinics, and Drs. Porter, Benedict and Lathrop were appointed a committee to arrange for them.

Dr. Crooks opened the discussion, saying that by skillfully treating the dental pulp, alveolar abscess may be avoided. He enumerated a large number of causes of inflammation of the dental pulp, and said that prophylactics were useful in its treatment. The inflammation ought to be stopped as soon as possible, and resolution induced that ulceration and abscess may be prevented. Even bleeding and leeching may be advantageously employed.

Dr. Douglass, of Romeo, was homœopathic in his views, and explained the remedies used by him.

Dr. Crooks did not approve of the homœopathic system.

Dr. Bancroft, of Romeo, advocated reducing inflammation of the periosteum by increasing it elsewhere—counter-irritation.

Dr. Holmes, of Battle Creek, thought that the simplest way of getting rid of the inflammation was the best and favored local applications, painting the gum, using aconite, iodine and belladonna.

Dr. Field, of Detroit, advocated the use of "mercurius vivus;" he had used it with great success in a large number of cases.

Dr. Stone uses cathartics, dovers powders, and slight doses of calomel, causing evacuation of the alimentary canal.

Dr. Corbin thought saline cathartics aided admirably, and with plethoric persons would deplete by scarification or bleeding.

Dr. Benedict thought creosote good for inflammation of the dental pulp ; but if the periosteum is affected, of course stronger remedies are to be resorted to.

The Association adjourned until 9 A. M., Thursday.

The Association met at 9 A. M., Thursday, Oct. 14.

Clinics being first in order, some time was spent in examining into the merits of an instrument for plugging teeth—called "Hyde's Pneumatic Dental Plugger"—exhibited by Dr. Jackson, of Ann Arbor. This machine, which weighs only twelve pounds, is composed of a treadle base, flexible pipe and plugger, or tool handle weighing only two ounces ; is capable of giving any number of blows, light or heavy, up to one thousand a minute; saves from one-fourth to one-half the ordinary time of filling; requires no assistant; gives less discomfort than any other mode of filling, and has the approval of the highest authorities in the Dental profession. Dr. Jackson performed an operation with this instrument upon Dr. Thomas, of Detroit, on the posterior cavity of the left superior first molar, filling it to his entire satisfaction, and much to the edification of the gentlemen present, who think very favorably of the instrument.

Dr. H. F. Lyster, of Detroit, by request, exhibited to the Association a cystic tumor, composed of a portion of the lower jaw which he successfully removed in April last, in the interior of the State, from a lady patient. The Doctor stated that from the left canine to the third molar the jaw had been expanded, on every side, to the size and thickness of a hen's egg. This change had been going on for seven years, and was supposed to have originated in irritation from an uncut tooth; and the fluid of the tumor had been evacuated several times for the relief of pain. Upon opening the tumor, after removal, a foreign body, about the size of a molar tooth, was discovered; which proved, upon examination, to be composed of salivary calculus. The operation for removal consisted in making an incision along the lower border of the tumor, and dissecting off the soft parts with the periosteum, and using a metacarpel saw and bone forceps just anterior to the canine and posterior to the third molar. The opening into the cavity of the mouth was made as late as possible to avoid hemorrhage into the pharynx. By keeping closely to the tumor, hemorrhage was avoided, and no vessel required a ligature. The dental artery was plugged in its bony canal. The dental nerve was discovered stretched across the interior of the tumor in its normal condition, but unsupported by any tissues. The tumor was filled with a mucous fluid of the appearance of saliva. The position of the chin and the contour of the face had been well preserved by moulding on binder's board, and by wiring the teeth on the sound side. A plate will be supplied with artificial teeth as soon as the parts have become strong and firm. The recovery of the patient was rapid and complete.

On motion of Dr. Field, the thanks of the Society were tendered to Dr. Lyster for the exhibition of this, to them, very interesting specimen, and for his practical remarks upon it.

Dr. Bancroft, of Romeo, read an interesting paper on the subject of "Dental caries and prophylactic measures for the same," its main purpose being a review of an article published in the February number of the *Dental Cosmos* for 1868. The writer of that article stated that the decay of teeth generally noticed by Dentists of late years, was due to the fermentation of food deposited in the act of mastication. Dr. Bancroft concurred with the writer in question on the matter of fermentation of food containing alcoholic or acid matter, but thought that a matter of minor importance when the plan of nature for the formation of bone was considered. A large per centage of the bone and cartilage is formed from mineral substance, and the durable portion or enamel is a cartilage which hardens. The gradations of the growth of cereals are as follows: Plant, earth, grain and then food; but in bolted flour a large proportion of the mineral substance is wasted—only 4 1-10 out of the 57 parts of bone making material, being retained, the rest being an utter waste of substances, which are essential to the formation of the bone. This, he thought, was especially the case where infants are deprived of the substances necessary in forming the bone. A large proportion of the teeth are formed when the child is teething, and if fine flour is used in such cases, as food for the child, its effects are soon apparent. He gave the proportions of mineral and vegetable matter contained in the teeth, and held that the unbolted flour, being the best producer of bone and cartilage, was much superior to the finer flour. He asked the Association to criticise his views and thoroughly discuss the opinions he held.

Dr. Corbin said that the husk part, or bran, contained the large part of the phosphates and carbonates of lime so much needed to properly form the osseous system, was a position generally admitted. One evil of too fine bolting is the fact that people often eat more fine wheat bread than the system demands, in order to supply nature with the requisite amount of mineral ingredients.

Dr. Crooks thought there was generally too much animal and too little earthy matter in the teeth; and in persons not beyond the meridian of life the deficiency might be remedied by a greater supply of the phosphates or carbonates of lime. The waste of every tissue in the body is supplied from the blood, and if that is supplied by food containing the requisite material, the remedy would be found. He had seen the femur of children so soft, from the deficiency of bone-making food, that it could be bent almost at right angles. He attached importance to a proper condition of the saliva; but if the urine was properly regulated, the saliva would generally be healthy. Dentists ought to be well acquainted with medicines to make such treatment a perfect success.

The following committees were appointed by the Chair to report next year on interesting subjects in Dentistry, they being instructed to correspond with the Chairman as to all interesting or unusual cases.

On Anatomy—Drs. Watling, E. S. Holmes and E. G Douglass.

Physiology—Drs. Thomas, Haukhurst and Corbin.

Chemistry—Drs. J. Robinson, G. P. Holmes and Geo. H. Mosher.

Hygiene—Drs. I. Douglass, Cutler and J. A. Harris.

Pathology—Drs. Stone, Warner and Knapp.

Therapeutics—Drs. Crook, Lathrop and Finch.

Operative Dentistry—Drs. Field, Bannister and Cummings.

Dental Education—Drs. Benedict, Bancroft and W. H. Jackson.

Artificial Dentures—Drs. Porter, Storms and Owen.

Instruments and Appliances—Drs. Smith, H. H. Jackson and J. C. Parker.

Dr. Corbin moved that the Secretary publish a circular containing a list of subjects and names of committee appointed to prepare papers on the same, and send to all active members of the Association. It is expected that members of committees will confer with each other, and also with others who may be interested in the subjects upon which they are, severally, to treat.

The following gentlemen were elected delegates to the National Dental Convention, which meets in August, 1870, at Nashville, Tennessee:

Drs. G. P. Holmes, of Battle Creek; Mosher, of Jackson; Corbin, of St. Johns; Bancroft, of Romeo; Smith, of Jackson; E. G. Douglass, of Lapeer; Storms, of Jonesville; T. A. White, of Battle Creek, and Finch, of Adrian.

The Committee on Clinics reported two operations by the "Pneumatic Plugger," one tooth extracted and four cases of filling, also the following resolution:

*Resolved*, That we have examined with special satisfaction Hyde's Pneumatic Dental Mallet, and recognize in it an instrument meeting a want in Dentistry hitherto unsupplied; and do cordially recommend it for its simplicity, facility, economy in time and labor, comfort both to the operator and to the patient, combined with the highest style of workmanship.

C. B. Porter,

H. Benedict,

J. Lathrop.

The report of the committee was accepted, and the resolution adopted.

The following gentlemen were appointed a committee to look into certain irregularities in Dental practice throughout the State, more particularly unprofessional conduct on the part of members of the Association:

Drs. Watling, of Ypsilanti; Smith, of Jackson, and Bannister, of Kalamazoo.

The following resolution, introduced by Dr. Holmes, of Battle Creek, was adopted:

*Resolved*, That Clinical exercises be hereafter excluded from the order of business of this Association.

The Convention, after passing a vote of thanks to the reporters of the city press and the proprietors of the Russell House, adjourned, to meet on the second Tuesday of October, 1870, at Jackson. GEO. H. MOSHER,
Secretary.

